# A lactate-related tSNE signature defines prognostic subtypes of bladder cancer and reveals LINC01094-mediated VIM stabilization in metastasis and drug resistance

**DOI:** 10.3389/fimmu.2025.1593523

**Published:** 2025-05-14

**Authors:** Pu Zhang, Hegan Zhang, Wanli Yu, Dage Fan, Yao Pan, Wei Zhuang, Fangzhen Cai, Qingliu He

**Affiliations:** ^1^ Department of Urology, Sichuan Provincial People’s Hospital, School of Medicine, University of Electronic Science and Technology of China, Chengdu, China; ^2^ Department of Gynecology, Quanzhou Women’s and Children’s Hospital, Quanzhou, China; ^3^ Department of Vascular Surgery, The First Affiliated Hospital of Chongqing Medical University, Chongqing, China; ^4^ Department of Pathology, The Second Affiliated Hospital of Fujian Medical University, Quanzhou, China; ^5^ Department of Obstetrics and Gynecology, The Second Affiliated Hospital of Fujian Medical University, Quanzhou, China; ^6^ Department of Urology, The Second Affiliated Hospital of Fujian Medical University, Quanzhou, China

**Keywords:** bladder cancer, tSNE score, lactate, metastasis, drug resistance

## Abstract

**Background:**

Bladder cancer (BLCA) is prone to metastasis and often shows poor responses to chemotherapy and immunotherapy. Investigating the underlying mechanisms of metastasis and drug resistance may therefore offer new therapeutic strategies for BLCA.

**Methods:**

Publicly available datasets were analyzed using consensus clustering and t-distributed stochastic neighbor embedding (tSNE) to characterize a lactate-related gene signature in BLCA. Gene set variation analysis (GSVA) was employed to assess signaling pathway activity, while immune cell infiltration in the tumor microenvironment (TME) was evaluated using single-sample gene set enrichment analysis (ssGSEA), the Estimation of Stromal and Immune cells in Malignant Tumors using Expression data (ESTIMATE), and CIBERSORT. RNA pull-down and RNA-binding protein immunoprecipitation (RIP) assays were then performed to confirm molecular interactions.

**Results:**

Two distinct BLCA subtypes were identified based on lactate-related gene expression, and a lactate-based tSNE score was constructed. This score demonstrated prognostic value and was integrated into a nomogram confirmed by a calibration curve. Functionally, higher tSNE scores correlated with immune- and inflammation-related pathways, as well as with immunotherapy efficacy in BLCA. Among candidate regulators identified, LINC01094 emerged as a key factor in BLCA metastasis and drug resistance. LINC01094 was predominantly localized in the cytoplasm and was upregulated in tumor tissues compared with adjacent normal tissues, acting as an unfavorable prognostic factor. *In vitro*, LINC01094 promoted metastasis and chemotherapy resistance, potentially by stabilizing VIM protein levels and inhibiting its ubiquitination.

**Conclusions:**

This comprehensive analysis of lactate-related genes reveals how this gene signature may shape the tumor microenvironment and affect BLCA patient prognosis. Additionally, our data suggest that targeting LINC01094 with antisense oligonucleotides (ASOs) could reduce BLCA cell metastasis and enhance their sensitivity to chemotherapy.

## 1 Introduction

Urothelial carcinoma comprises malignancies originating from the urothelium, including renal pelvis cancer, ureteral cancer, bladder cancer (BLCA), and urethral cancer. Among these, BLCA is the most common subtype of urothelial carcinoma and ranks fourth in incidence among cancers in certain populations (1). Muscle-invasive bladder cancer (MIBC) accounts for approximately 30% of all BLCA cases and typically progresses rapidly with high rates of recurrence and drug resistance (2, 3). Over time, local treatment modalities (e.g., surgery and radiotherapy) and systemic therapies (e.g., chemotherapy and immunotherapy) have incrementally improved BLCA outcomes. Nonetheless, although the standard first-line chemotherapy regimen containing gemcitabine plus cisplatin is generally effective in BLCA, the overall survival (OS) for patients with T3-T4 and/or N+ disease remains below 30% (4). Immunotherapy agents, such as nivolumab, have yielded promising benefits as adjuvant treatments for BLCA patients following radical cystectomy (5) and can also be administered in neoadjuvant settings, with or without chemotherapy, prior to surgery (6). However, accurately identifying those patients who will benefit most from immunotherapy and clarifying the mechanisms underlying chemotherapy resistance remain significant challenges in BLCA management.

The Warburg effect posits that cancer cells rely primarily on glycolysis rather than aerobic respiration, even in the presence of sufficient oxygen. This process generates excess lactate, which accumulates in the tumor microenvironment (TME) (7). Although lactate is traditionally viewed as a terminal metabolite of glycolysis, accumulating evidence indicates that lactate can participate in critical biological functions. Notably, during tumor initiation and progression, lactate modulates the TME and can facilitate immune evasion (8). Several studies have confirmed that lactate activates intracellular signaling pathways that regulate cellular behavior and function within the TME (9). For instance, lactate positively influences the metabolic activity of regulatory T (Treg) cells, thereby enhancing their immunosuppressive capacity (10), and it also fosters the polarization of macrophages toward an M2-like phenotype, promoting angiogenesis, tissue remodeling, tumor growth, and invasion (11). Hence, establishing a molecular classification of BLCA based on lactate-related genes may help illuminate the tumor’s biological features governed by lactate. Additionally, constructing a lactate-based scoring system could potentially predict immunotherapy outcomes and guide more personalized treatment approaches.

Previous research has demonstrated that subtype classifications grounded in lactate-related genes can yield new methods for risk stratification in colorectal and hepatocellular carcinomas, thereby improving prognosis prediction and treatment efficacy (12, 13). Recently, several gene signature–based risk scores have demonstrated significant prognostic value and predictive capability for BLCA treatments (14–16). However, a comparable classification system and risk score specifically derived from lactate-related genes have not yet been constructed for BLCA. Consequently, using publicly available BLCA datasets, we first applied consensus clustering based on lactate-related gene signatures to identify key signaling pathways potentially regulated by lactate. By employing a machine learning strategy known as t-distributed stochastic neighbor embedding (tSNE), we then developed a lactate-related tSNE score to quantify inter-patient variation. Through correlation analyses, we identified a long non-coding RNA (lncRNA), LINC01094, which exhibited the strongest association with the tSNE score and effectively captured key clinical and biological features. LncRNAs have been widely investigated in BLCA due to their potent regulatory functions and clinical relevance (17–19). Therefore, clarifying the biological role and clinical significance of LINC01094 may provide insights that span the entire therapeutic trajectory in BLCA.

In this study, we used fluorescence *in situ* hybridization (FISH) and quantitative real-time PCR (qRT-PCR) to validate the expression level of LINC01094 in BLCA tissues and cell lines. Mechanistic assays, including RNA pull-down and RNA-binding protein immunoprecipitation (RIP), confirmed the direct interaction between LINC01094 and VIM. Furthermore, after manipulating LINC01094 expression (either ectopically overexpressing or knocking it down), we investigated its impact on metastasis and chemotherapy resistance in BLCA cell lines. Our findings not only suggest that LINC01094 plays an important role in BLCA progression but also highlight a potential avenue for improving treatment strategies.

## 2 Materials and methods

### 2.1 Deriving a tSNE-based scoring system and assessing its prognostic significance

In order to investigate how lactate-related genes cluster within BLCA, we employed nonnegative matrix factorization (NMF) to classify patients into subgroups. We selected two clusters based on a notable decline in the cophenetic coefficient. Building on a prior approach used for principal component analysis (PCA)–derived scores, we generated two tSNE components (tSNE1 and tSNE2) per sample from the lactate-related gene expression matrix (gene i). We then defined the tSNE score as the sum of those two components:


tSNE score=Σ(tSNE1i+tSNE2i)

Subsequently, we applied the “rms” package in R to develop a prognostic nomogram that integrated clinical risk parameters with the tSNE score. We used calibration curves to gauge how effectively the nomogram predicted patient survival. Lastly, to confirm the nomogram’s reliability, four external BLCA cohorts were employed to compute tSNE scores and evaluate their prognostic relevance.

### 2.2 Cell culture procedures and key reagents

We obtained four BLCA cell lines (5637, UM-UC-3, T24, and EJ) and one normal uroepithelial line (SV-HUC-1) from the Cell Bank of the Chinese Academy of Sciences (Shanghai, China). These cells were cultured in RPMI 1640 medium (Gibco, NY, USA) supplemented with 10% fetal bovine serum (ScienCell, CA, USA) and 1% penicillin–streptomycin (HyClone, UT, USA), maintained at 37°C under a humid environment with 5% CO_2_. Each cell line’s identity was authenticated by short tandem repeat analysis, and regular screenings confirmed they were free from mycoplasma. Actinomycin D (ActD, S8964) and MG132 (S2619) were sourced from Selleck (Houston, TX, USA). The key primary antibodies used were anti-VIM, anti-GAPDH, and anti-ubiquitin (Cell Signaling Technology, USA).

### 2.3 Fluorescence *in situ* hybridization

Biotin-labeled probes targeting LINC01094 were designed and synthesized by RiboBio (Guangzhou, China). FISH assays were conducted using the Fluorescent *In Situ* Hybridization Kit (Ribo) according to the manufacturer’s instructions. Briefly, T24 or 5637 cells, as well as tissue microarrays, were hybridized with the probes and then counterstained with DAPI. Images were acquired on a Leica SP5 confocal microscope (Leica Microsystems, Wetzlar, Germany).

### 2.4 RNA-binding protein immunoprecipitation

RIP experiments were carried out using the EZ-magna RIP kit (Millipore, Billerica, MA, USA), following the manufacturer’s protocol. A rabbit anti-VIM antibody or rabbit IgG (as a control) was used for immunoprecipitation. The coprecipitated RNAs were subsequently examined by qRT-PCR.

### 2.5 qRT-PCR analysis and primer sequences

Total RNA was extracted using TRIzol reagent (Invitrogen, USA), and complementary DNA (cDNA) was generated with a High-Capacity cDNA Reverse Transcription Kit (Takara, Japan) according to the supplier’s protocols. Quantification of LINC01094 transcripts was conducted with the TB Green kit (Takara, Japan), employing GAPDH as an internal control via the 2^−ΔΔCT calculation. The primer sequences were:

LINC01094 forward: 5′-GGCCACCAAGTCTGCAATTCTCC-3′
LINC01094 reverse: 5′-TCCCAGTGCTCCCTCTTCCTTTC-3′
GAPDH forward: 5′-ACCCAGAAGACTGTGGATGG-3′
GAPDH reverse: 5′-CAGTGAGCTTCCCGTTCAG-3′

### 2.6 Transwell migration and invasion assay

Cell migration and invasion were assessed using Transwell chambers (Millipore, Billerica, USA). For migration assays, 5 × 10^4 transfected cells were seeded into the upper chamber containing serum-free medium, while RPMI 1640 medium supplemented with 10% FBS was placed in the lower chamber. For invasion assays, the upper side of the membrane was precoated with Matrigel (BD Biosciences, Franklin Lakes, NJ, USA), and the procedure otherwise mirrored the migration assay. After 24 hours, cells on the lower surface were fixed, stained with crystal violet, and counted under a light microscope.

### 2.7 Cell counting kit-8 assay

Cell proliferation was quantified using the CCK-8 assay according to the manufacturer’s protocol. In brief, 5 × 10^3 BLCA cells were seeded into each well of a 96-well plate. At designated time points, 10 μL of CCK-8 solution was added, followed by incubation for 1–2 hours. The absorbance at 450 nm was recorded with a microplate reader. Additional details on these and other experimental procedures are provided in the **Supplementary Materials**.

## 3 Results

### 3.1 Biological characteristics of BLCA subtypes clustered by lactate-related genes

**Figure 1:**
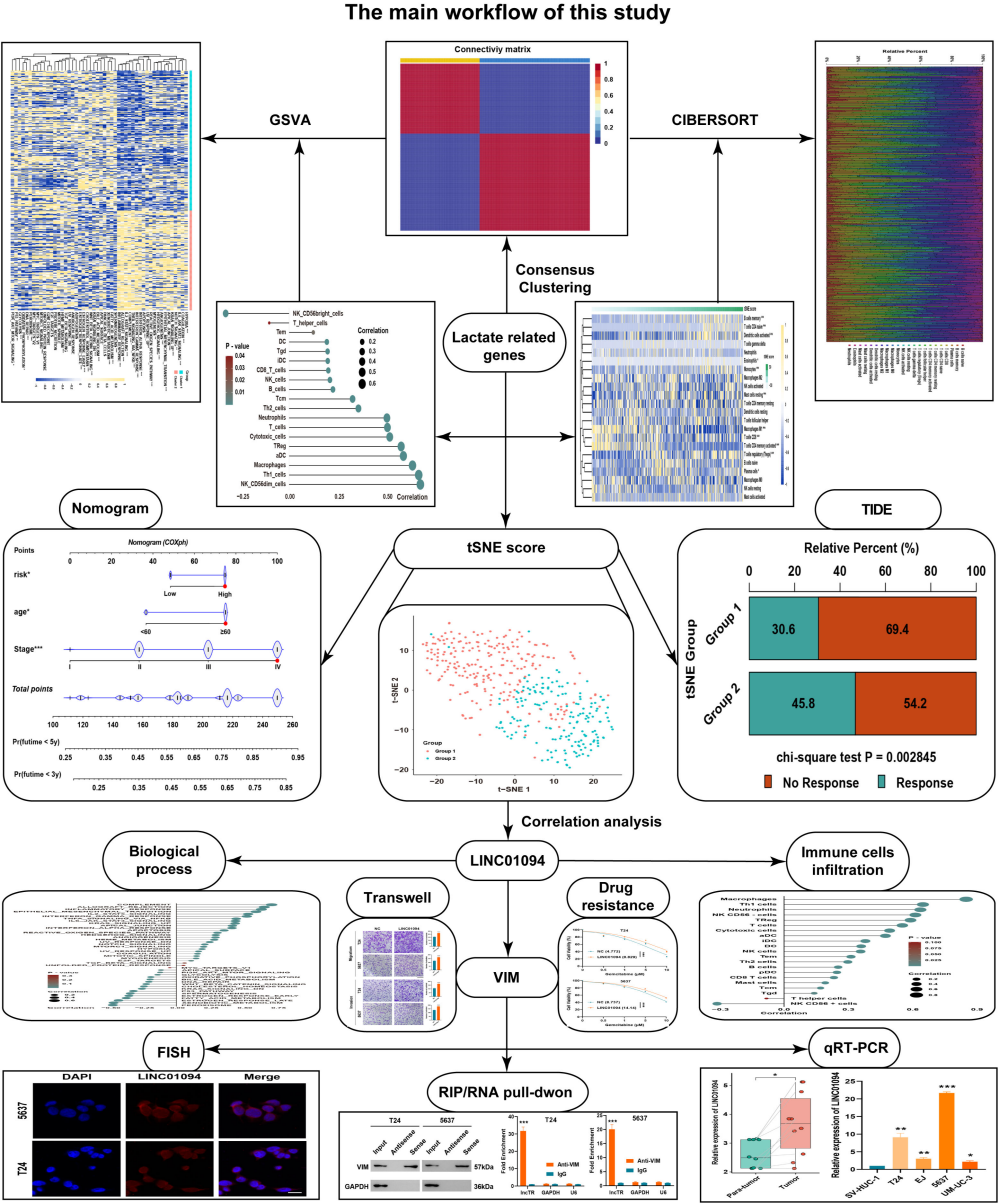
The overall workflow of this study. A schematic diagram illustrating the study design, including data retrieval, consensus clustering based on lactate-related genes, tSNE score construction, and subsequent experimental validations.

**Figure 2:**
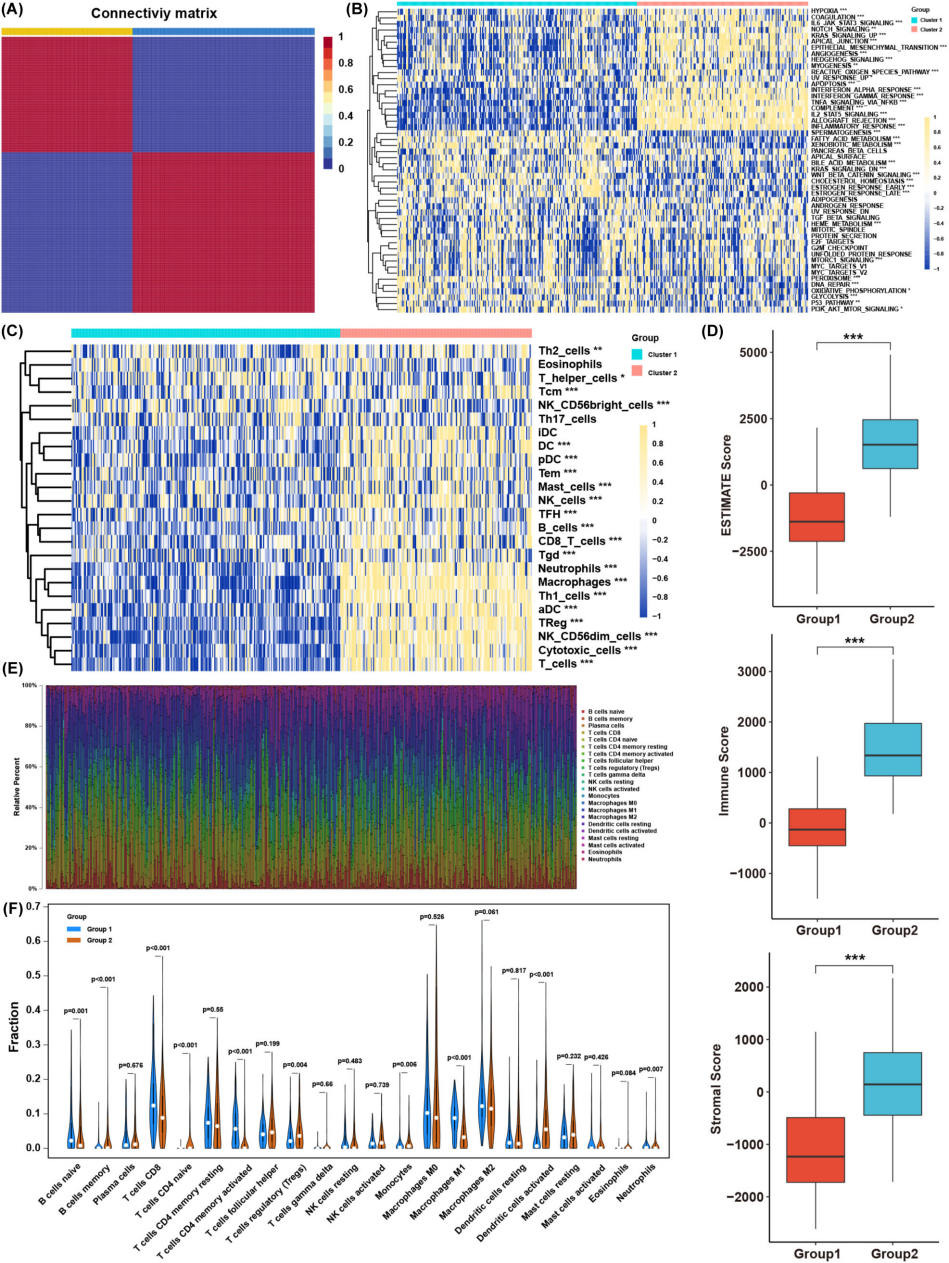
Biological characteristics of BLCA subtypes clustered by lactate-related genes. **(A)** Consensus clustering results of lactate-related genes in the TCGA BLCA cohort. **(B)** Comparison of GSVA enrichment scores between the two BLCA clusters. **(C)** Infiltration levels of immune cells, as determined by ssGSEA, in each cluster. **(D)** ESTIMATE scores (immune + stromal) in the two clusters. **(E)** The proportions of various immune cell types based on CIBERSORT analysis. **(F)** Distribution of specific immune cell subsets between the two clusters. *p< 0.05, **p< 0.01, ***p< 0.001.

A total of 206 lactate-related genes were retrieved from a previous study (12), as listed in **Supplementary Table S1**. The overall workflow of this study is shown in **Figure 1**. To better characterize these lactate-related genes, we performed consensus clustering on BLCA samples in the TCGA database (**Supplementary Figure S1**). Based on the cophenetic metric, patients were stratified into two distinct groups with specific gene expression patterns (**Figure 2A**). Gene set variation analysis (GSVA) revealed significant differences in pathway activation between the two clusters (**Figure 2B**), suggesting that variations in lactate-related gene expression underlie diverse biological functions. Specifically, immune- and inflammation-related pathways (e.g., interferon- and interleukin-regulated signaling) were highly enriched in cluster 2, whereas metabolic-related pathways (e.g., fatty acid, xenobiotic, bile acid metabolism, and glycolysis) were predominantly enriched in cluster 1. These observations imply that accumulated lactate may be linked to both immune activity and metabolic processes in the tumor microenvironment (TME).

Single-sample gene set enrichment analysis (ssGSEA) confirmed that most immune cell types were abundantly infiltrated in cluster 2, although both immunosuppressive and immune-activated populations were present (**Figure 2C**). Consistently, ESTIMATE analysis showed that cluster 2 had higher immune and stromal scores (**Figure 2D**). To further compare immune infiltration between the two clusters, we applied CIBERSORT to estimate immune cell proportions (**Figure 2E**). B cells, T cells, macrophages, dendritic cells, and neutrophils differed significantly between clusters. Although cluster 2 exhibited higher overall immune infiltration, the proportion of regulatory T cells (Tregs) was also elevated, whereas CD8+ T cells and M1 macrophages were decreased (**Figure 2F**). These findings indicate that lactate-related gene signatures strongly associate with immune- and inflammation-related pathways, as well as with immune cell infiltration in the TME. Nevertheless, the precise balance between immune activation and suppression in the context of lactate-related gene expression warrants further investigation.

### 3.2 Construction of a lactate-related tSNE score for BLCA

**Figure 3:**
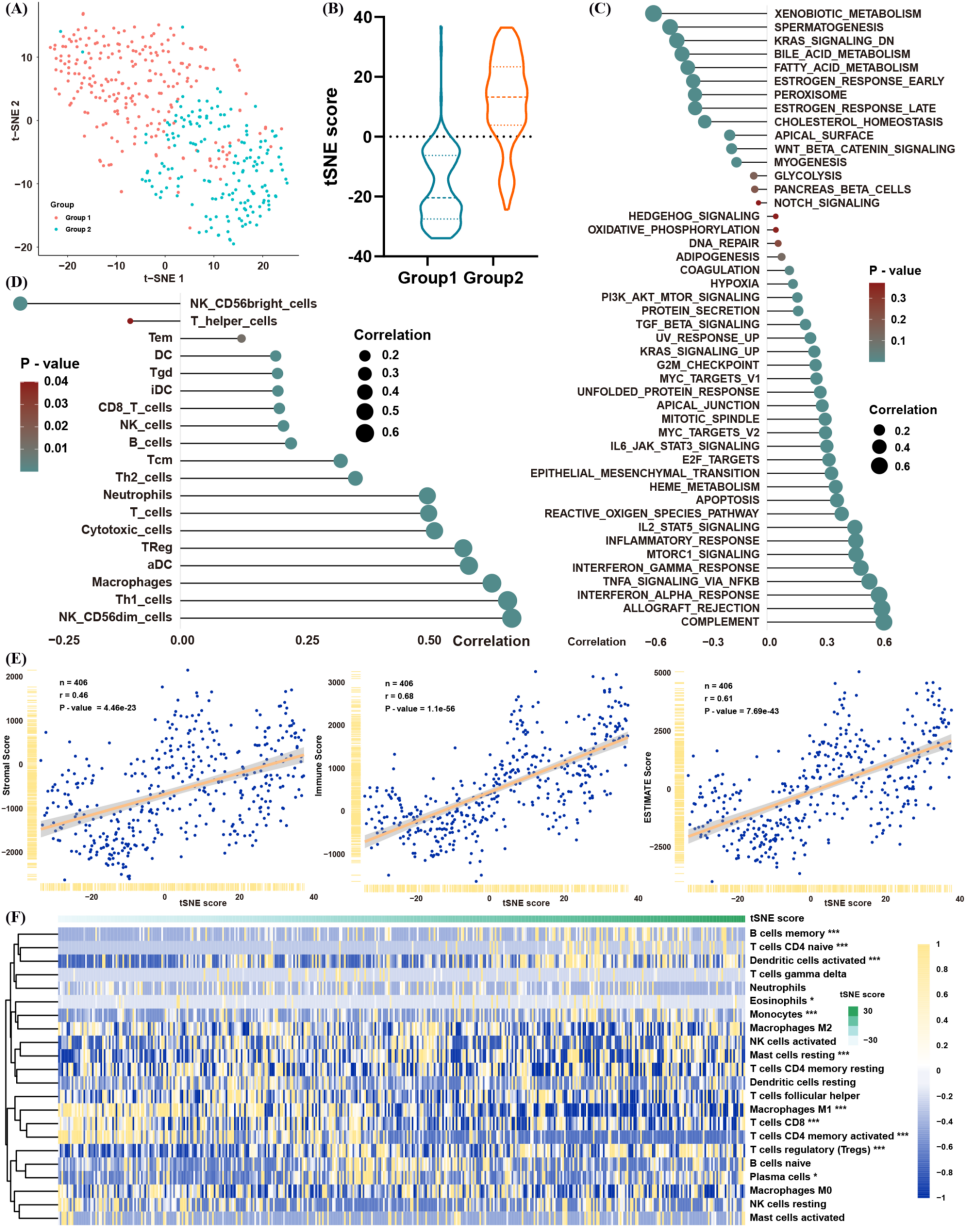
Construction of a lactate-related tSNE score for BLCA. **(A)** tSNE plot showing separation of the two BLCA clusters. **(B)** Distribution of tSNE scores between the clusters. **(C)** Spearman correlation between the tSNE score and GSVA pathway enrichment. **(D)** Spearman correlation between the tSNE score and infiltration levels of immune cells (ssGSEA). **(E)** Spearman correlation between the tSNE score and ESTIMATE scores (immune/stromal). **(F)** Spearman correlation between the tSNE score and proportions of immune cells in ssGSEA. *p< 0.05, **p< 0.01, ***p< 0.001.

Considering the heterogeneity observed in the two lactate-defined BLCA subtypes, we generated a tSNE score to quantify individual variations in lactate-related gene expression. Two tSNE dimensions effectively distinguished the two BLCA clusters (**Figure 3A**), and each cluster displayed distinct tSNE score distributions (**Figure 3B**). To assess the functional relevance of the tSNE score, we correlated the enrichment scores of GSVA pathways with each patient’s tSNE score (**Figure 3C**). The tSNE score was positively associated with immune- and inflammation-related pathways (e.g., interferon and interleukin signaling), as well as tumor progression and metastasis pathways (e.g., TGF-β, MYC, E2F, and EMT). Conversely, it was negatively associated with metabolic-related pathways, mirroring the features of cluster 1.

In line with these results, the tSNE score was positively correlated with multiple immune cell populations—encompassing both immunosuppressive and anti-tumor subsets (**Figure 3D**)—and was also positively correlated with immune and stromal scores in the ESTIMATE analysis (**Figure 3E**). CIBERSORT likewise confirmed that patients with higher tSNE scores tended to have greater proportions of Tregs and fewer CD8+ T cells and M1 macrophages (**Figure 3F**). Collectively, these data suggest that the tSNE score robustly reflects the biological distinctions initially observed between the two lactate-based BLCA clusters, providing a powerful tool for further analyses.

### 3.3 Clinical application of the tSNE score and validation of its prognostic predictive capability in BLCA

**Figure 4:**
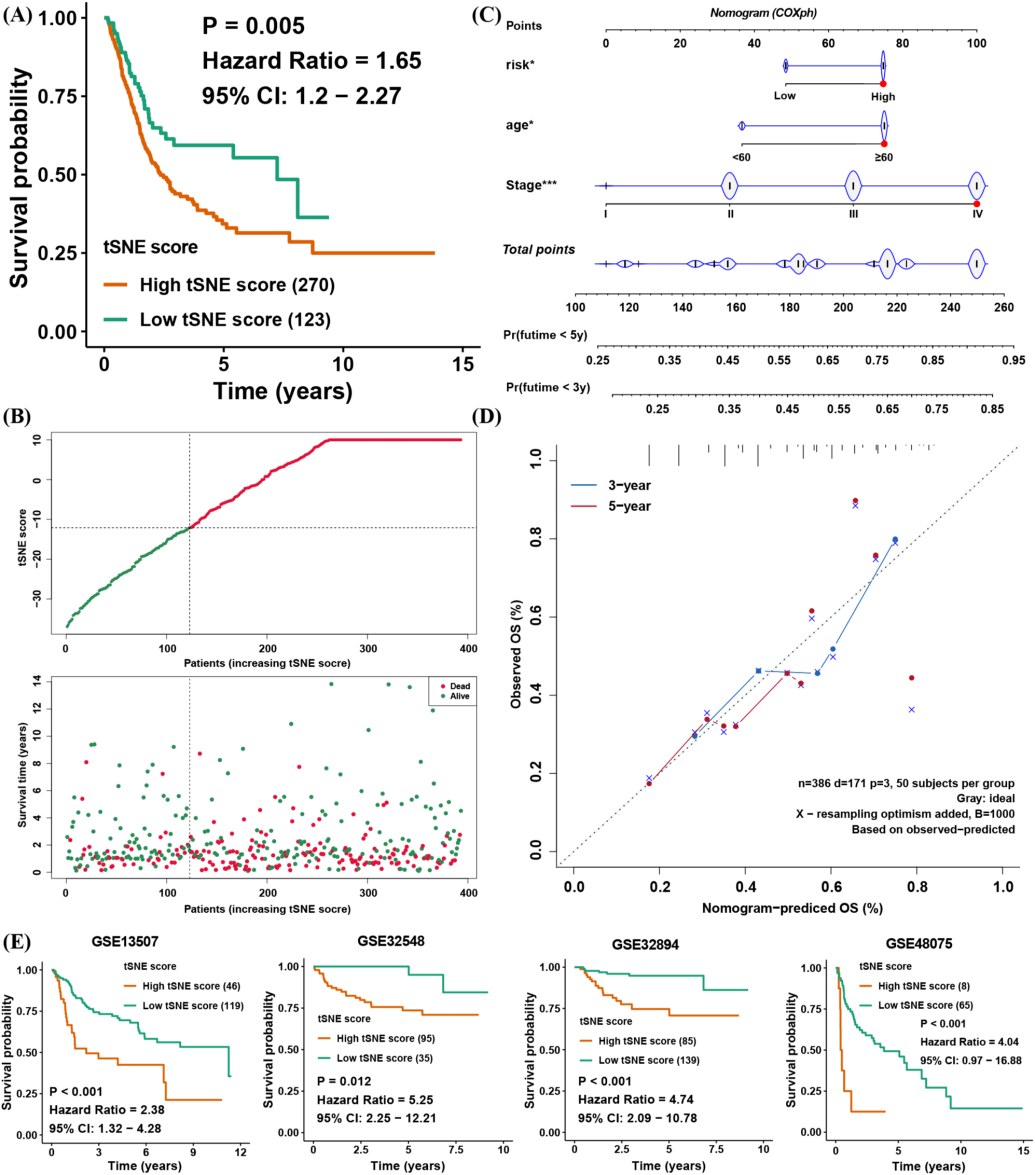
Clinical application of the tSNE score and validation of its prognostic capability for BLCA. **(A)** Kaplan–Meier survival analysis comparing high and low tSNE score groups. **(B)** Distribution of tSNE scores and patient survival status. **(C)** Nomogram integrating age, stage, and tSNE score to predict 3- and 5-year overall survival. **(D)** Calibration curves evaluating the nomogram’s predictive performance at 3 and 5 years in TCGA BLCA. **(E)** Validation of tSNE score prognostic value in four external BLCA datasets. *p< 0.05, ***p< 0.001.

The above findings illustrate the biological and TME-related importance of the tSNE score. To evaluate its prognostic utility, we performed survival analyses, which demonstrated that the tSNE score indeed functioned as a risk factor for BLCA patients (**Figures 4A, B**). To facilitate potential clinical use, we then built a nomogram combining age, stage, and the tSNE score to predict 3- and 5-year survival probabilities (**Figure 4C**). Calibration curves showed good agreement between the nomogram’s predictions and actual survival outcomes at both time points (**Figure 4D**).

To further verify the prognostic value of the tSNE score, four additional BLCA cohorts with transcriptomic data and survival information were analyzed. In each cohort, patients with higher tSNE scores consistently exhibited worse overall survival (**Figure 4E**). These results confirm that the tSNE score is a stable and reliable predictor of BLCA prognosis, potentially aiding clinical decision-making.

### 3.4 Investigating the biological and immune features linked to the tSNE score

**Figure 5:**
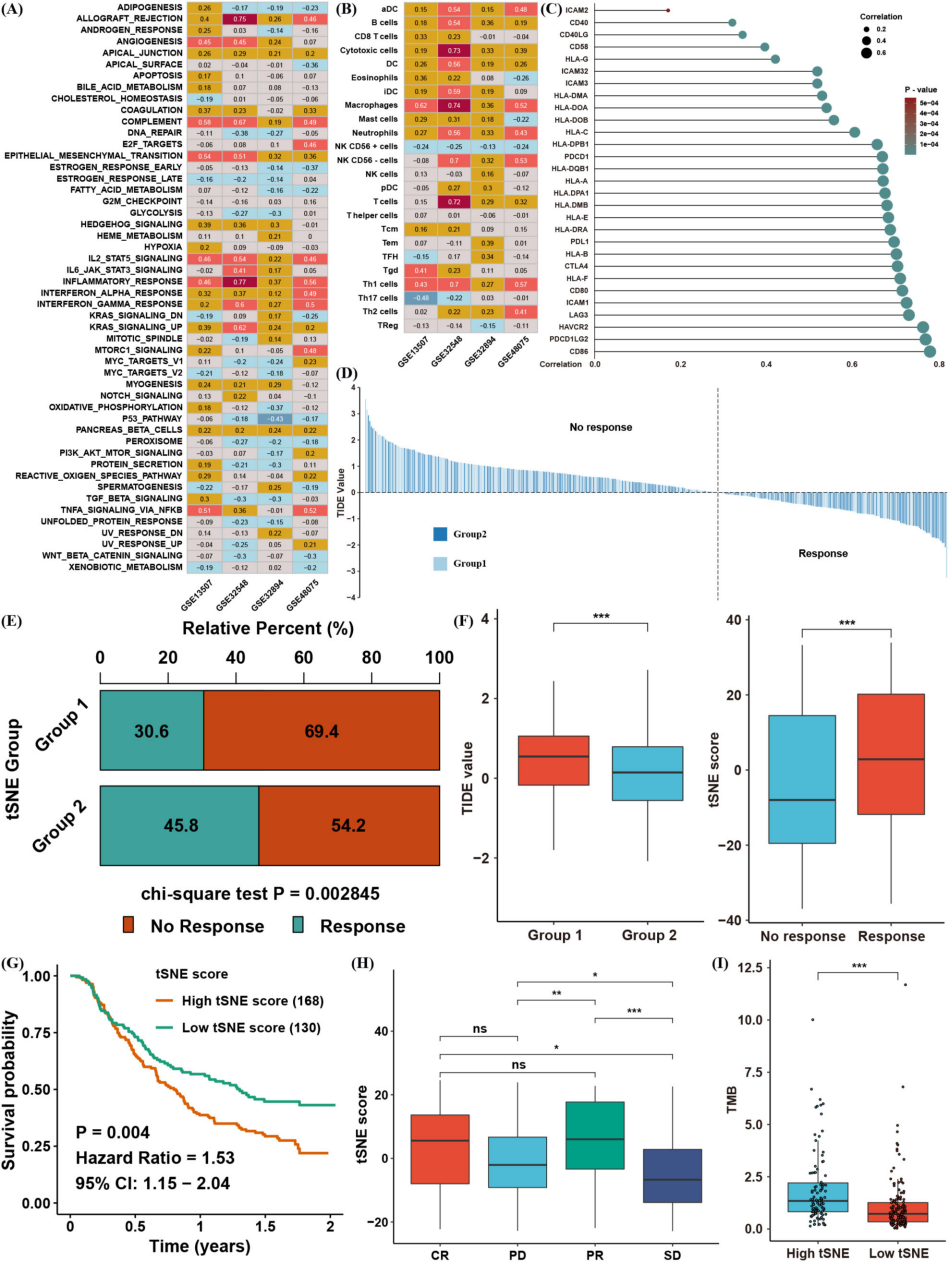
Biological and immune-related features associated with the tSNE score. **(A)** Spearman correlation between the tSNE score and GSVA enrichment scores across four independent BLCA datasets. **(B)** Spearman correlation between the tSNE score and tumor microenvironment (TME) infiltration levels. **(C)** Spearman correlation between the tSNE score and expression of MHC, costimulatory, and adhesion molecules. **(D)** TIDE values for TCGA BLCA samples, stratified by tSNE score. **(E)** Chi-square analysis comparing immunotherapy response between different tSNE score groups. **(F)** Distribution of TIDE values in each BLCA cluster and tSNE scores by immunotherapy response group. **(G)** Kaplan–Meier survival analysis of tSNE score in the IMvigor210 cohort. **(H)** Distribution of tSNE scores among complete response (CR), partial response (PR), progressive disease (PD), and stable disease (SD) groups. **(I)** Distribution of tumor mutation burden (TMB) in different tSNE score groups. *p< 0.05, **p< 0.01, ***p< 0.001. ns, not significant.

Next, we extended our validation of the tSNE score’s biological significance across four independent BLCA datasets (**Figure 5A**). The tSNE score was consistently and positively correlated with pathways involved in allograft rejection, apical junction, complement, EMT, IL2/STAT5, interferon-α/β, inflammatory responses, KRAS signaling, and pancreas-β cell–related signaling. No pathway showed a consistently negative correlation with the tSNE score in these datasets. We then examined immune cell infiltration levels in the TME to identify which cell populations were most correlated with the tSNE score. Notably, most immune cell infiltration levels were positively correlated with the tSNE score, except for CD56+ NK cells, which showed a consistent negative correlation (**Figure 5B**). Moreover, the tSNE score was positively correlated with various MHC molecules, costimulatory molecules, and adhesion molecules. Of particular interest, immune checkpoints such as PD1, PD-L1, and CTLA4 were also positively correlated with the tSNE score, suggesting an immunosuppressive TME that could benefit from immunotherapy (**Figure 5C**).

To explore this possibility, we performed TIDE analysis to predict immunotherapy responses in TCGA BLCA patients (**Figure 5D**). Patients were categorized as responders or non-responders based on TIDE values, and the chi-square test indicated that cluster 2 patients (those associated with higher tSNE scores) had a superior response rate (**Figure 5E**). Indeed, cluster 2 displayed a significantly lower TIDE value, consistent with an increased likelihood of immunotherapeutic benefit (**Figure 5F**). To strengthen these findings, we evaluated an anti–PD-L1 cohort (IMvigor210) of urinary carcinoma. Patients with high tSNE scores had worse overall survival (**Figure 5G**), yet the tSNE score in complete (CR) and partial response (PR) groups was significantly higher than in progressive disease (PD) or stable disease (SD) groups (**Figure 5H**). Notably, patients with elevated tumor mutation burden (TMB) also tended to have higher tSNE scores and potentially better responses to anti–PD-L1 therapy (**Figure 5I**). Taken together, these observations confirm that lactate-related gene signatures correlate strongly with the TME and immunotherapy response, and the tSNE score may serve as an effective biomarker for predicting immunotherapy outcomes in BLCA.

### 3.5 Identification of a candidate gene associated with the tSNE score

**Figure 6:**
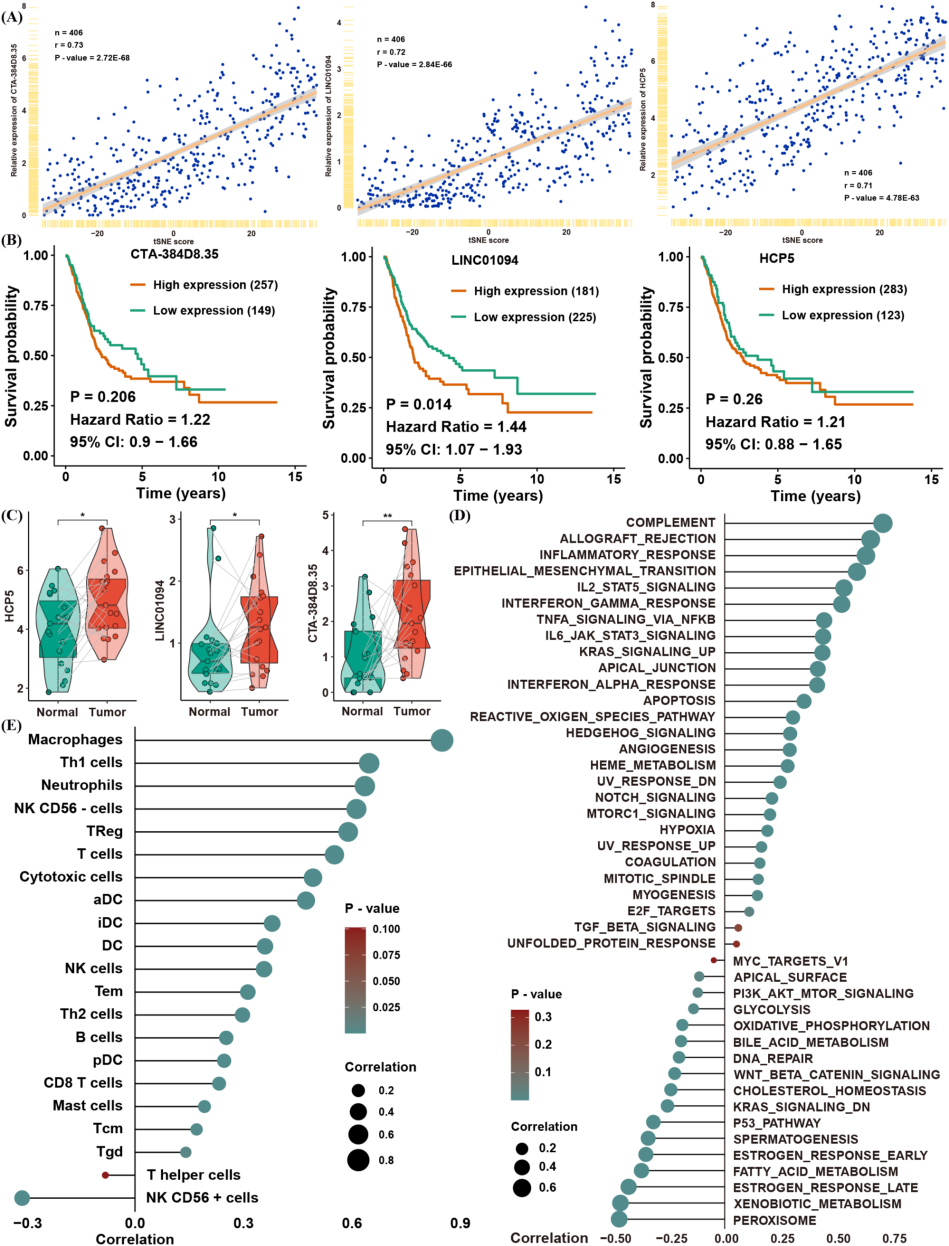
Identification of a candidate gene associated with the tSNE score. **(A)** Correlation of CTA-384D8.35, LINC01094, and HCP5 expression with the tSNE score. **(B)** Kaplan–Meier survival analysis of CTA-384D8.35, LINC01094, and HCP5 in the TCGA BLCA dataset. **(C)** Box plots showing expression levels of the three lncRNAs in paired normal vs. BLCA tissues. **(D)** Spearman correlation between LINC01094 expression and GSVA pathway enrichment scores. **(E)** Spearman correlation between LINC01094 expression and immune cell infiltration levels. *p< 0.05, **p< 0.01.

To uncover lncRNAs potentially implicated in lactate regulation, we correlated lncRNA expression levels with the tSNE score in TCGA (**Supplementary Table S2**). A total of 1,601 lncRNAs showed significant correlations; among them, only three—CTA-384D8.35, LINC01094, and HCP5—had absolute correlation coefficients exceeding 0.7 (**Figure 6A**). Further survival analysis confirmed that only LINC01094 significantly served as a risk factor for BLCA (**Figure 6B**), despite all three lncRNAs being differentially expressed in tumor versus normal tissues (**Figure 6C**).

**Figure 7:**
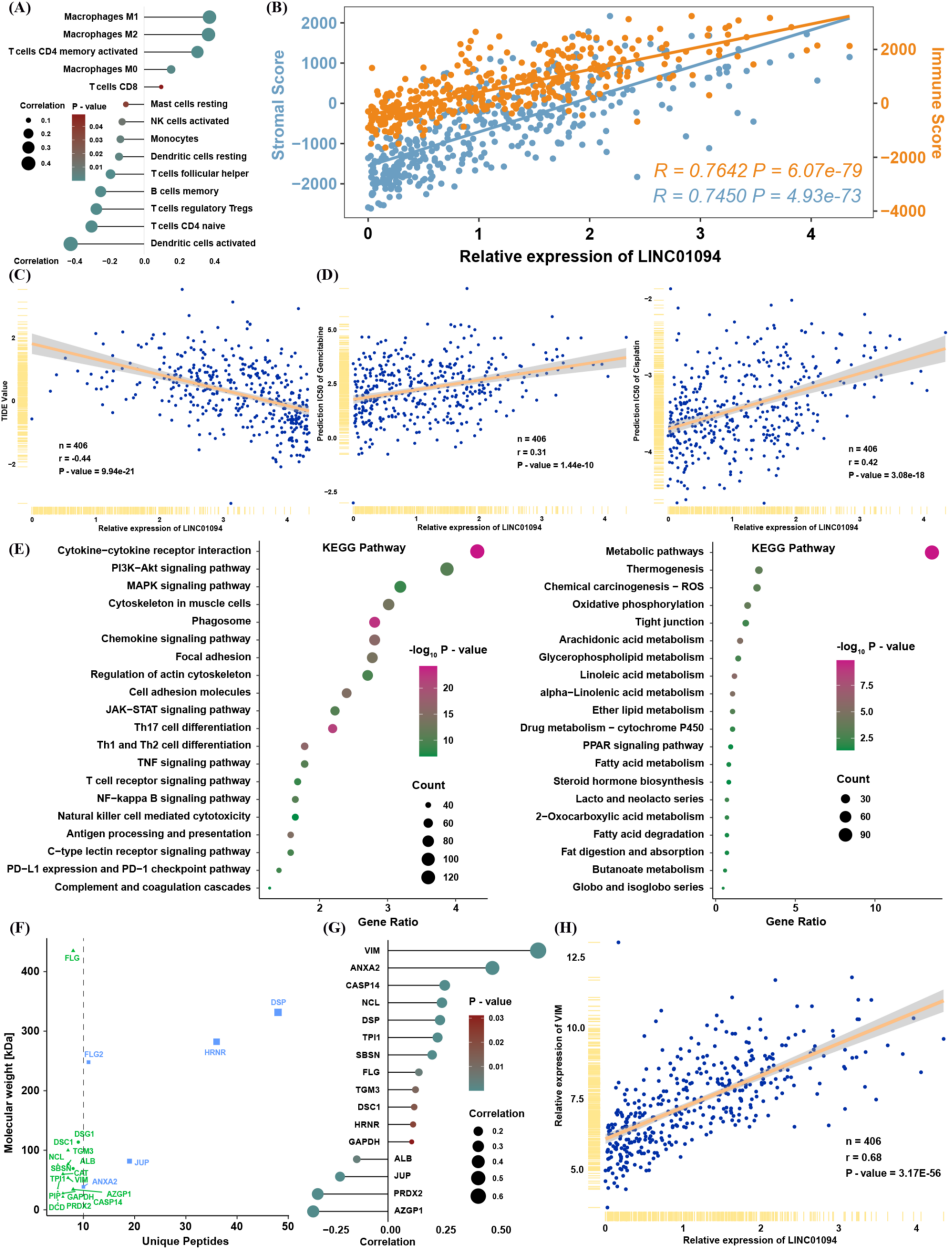
Biological and immune-related features of LINC01094. **(A)** Spearman correlation between LINC01094 expression and proportions of immune cells. **(B)** Spearman correlation between LINC01094 expression and ESTIMATE scores. **(C)** Correlation between LINC01094 expression and TIDE values. **(D)** Correlation between LINC01094 expression and predicted IC50 for gemcitabine and cisplatin. **(E)** KEGG pathway analysis of genes positively (left) or negatively (right) correlated with LINC01094 expression. **(F)** Interactive proteins co-precipitated by probes targeting LINC01094, identified via mass spectrometry. **(G)** Spearman correlation between LINC01094 and the indicated candidate proteins in TCGA. **(H)** Spearman correlation between LINC01094 and VIM mRNA expression in TCGA.

To validate the relationship between LINC01094 expression and the tSNE score, we found that LINC01094 was also significantly correlated with pathways that were themselves associated with the tSNE score (**Figure 6D**). Notably, macrophages emerged as the most relevant immune cell population linked to LINC01094 expression, suggesting a potential role of LINC01094 in macrophage polarization within the TME (**Figure 6E**). In line with this hypothesis, CIBERSORT analysis revealed that LINC01094 was highly correlated with M0, M1, and M2 macrophages (**Figure 7A**) and was positively associated with both immune and stromal scores from ESTIMATE (**Figure 7B**). Additional correlation analyses suggested that patients with higher LINC01094 expression might be more likely to benefit from immunotherapy (**Figure 7C**).

We also investigated whether LINC01094 expression is involved in chemotherapy resistance by analyzing predicted IC50 values for cisplatin and gemcitabine in TCGA (**Figure 7D**). The strong positive correlation between LINC01094 and IC50 values for these drugs implies that LINC01094 may contribute to chemoresistance. Moreover, functional enrichment of mRNAs significantly correlated with LINC01094 (|R| > 0.3, P< 0.05) indicated that positively correlated genes were enriched in immune- and tumor progression–related pathways, while negatively correlated genes were enriched in metabolic pathways (**Figure 7E**).

To further clarify LINC01094’s function in BLCA, we reanalyzed a mass spectrometry dataset from a public RNA pull-down study (20). Among the 17 proteins identified by at least five unique peptides (**Figure 7F**), VIM (an EMT-related molecule) displayed the highest correlation with LINC01094 expression in TCGA (**Figures 7G–H**). These findings prompted us to focus on the molecular mechanisms by which LINC01094 may regulate BLCA progression through VIM.

### 3.6 LINC01094 serves as a risk factor by maintaining VIM protein stability

**Figure 8:**
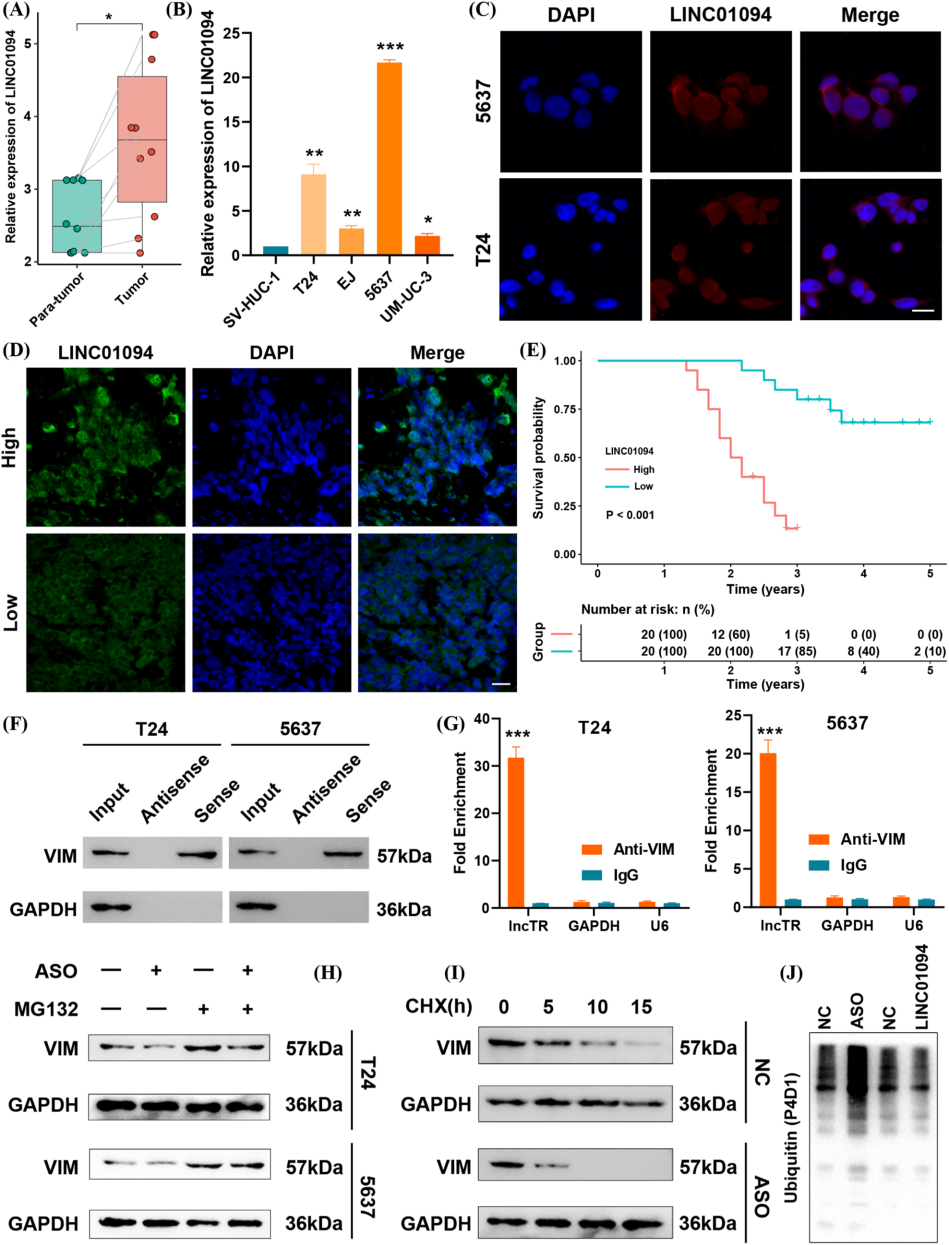
LINC01094 functions as a risk factor by maintaining VIM protein stability. **(A)** Baseline expression of LINC01094 in tumor vs. para-tumor tissues. **(B)** LINC01094 expression in BLCA cell lines compared with a normal uroepithelial cell line. **(C)** FISH assay showing LINC01094 localization in BLCA cells. scale bar 10um. **(D)** FISH assay of LINC01094 in BLCA tissues. scale bar 20um. **(E)** Kaplan–Meier survival analysis for LINC01094 expression in BLCA patients. **(F)** RNA pull-down assay verifying interaction between LINC01094 and VIM. **(G)** RIP assay results confirming LINC01094 binding to VIM. **(H)** Western blot analysis of VIM under LINC01094 knockdown ± MG132 treatment in T24 and 5637 cells. **(I)** Cycloheximide (CHX) chase assay assessing VIM half-life after LINC01094 knockdown. **(J)** Polyubiquitination levels of VIM protein upon LINC01094 overexpression or knockdown. *p< 0.05, **p< 0.01, ***p< 0.001.

Based on the above evidence, we measured LINC01094 mRNA levels in BLCA tissues and cell lines by qRT-PCR. LINC01094 expression was significantly higher in tumor samples than in adjacent normal tissues in our cohort of ten matched pairs (**Figure 8A**), and it was also elevated in BLCA cell lines compared with normal uroepithelial cells (**Figure 8B**). Retrieval of subcellular localization data from the lncATLAS database indicated that LINC01094 is primarily located in the cytoplasm (**Supplementary Figure S2**), which was further confirmed by FISH analysis (**Figure 8C**). Additionally, high LINC01094 expression correlated with shorter overall survival in BLCA (**Figures 8D–E**).

To investigate the underlying mechanism, we reexamined public mass spectrometry data and performed an RNA pull-down in BLCA cells. Western blotting revealed a specific interaction between LINC01094 and VIM (**Figure 8F**). A subsequent RIP assay confirmed the direct binding of LINC01094 to VIM (**Figure 8G**). We then tested whether LINC01094 modulates VIM degradation through the ubiquitin–proteasome pathway. Notably, when proteasome-mediated degradation was inhibited by MG132, knocking down LINC01094 no longer affected VIM levels (**Figure 8H**). Furthermore, the half-life of VIM protein was extended when LINC01094 was overexpressed and shortened when LINC01094 was knocked down (**Figure 8I**). Consistent with these observations, upregulated LINC01094 suppressed VIM polyubiquitination, whereas antisense oligonucleotides (ASOs) targeting LINC01094 enhanced it (**Figure 8J**). Collectively, our findings indicate that LINC01094 binds to VIM and stabilizes its protein levels in BLCA.

### 3.7 LINC01094 promotes metastasis and chemotherapy resistance in BLCA

**Figure 9:**
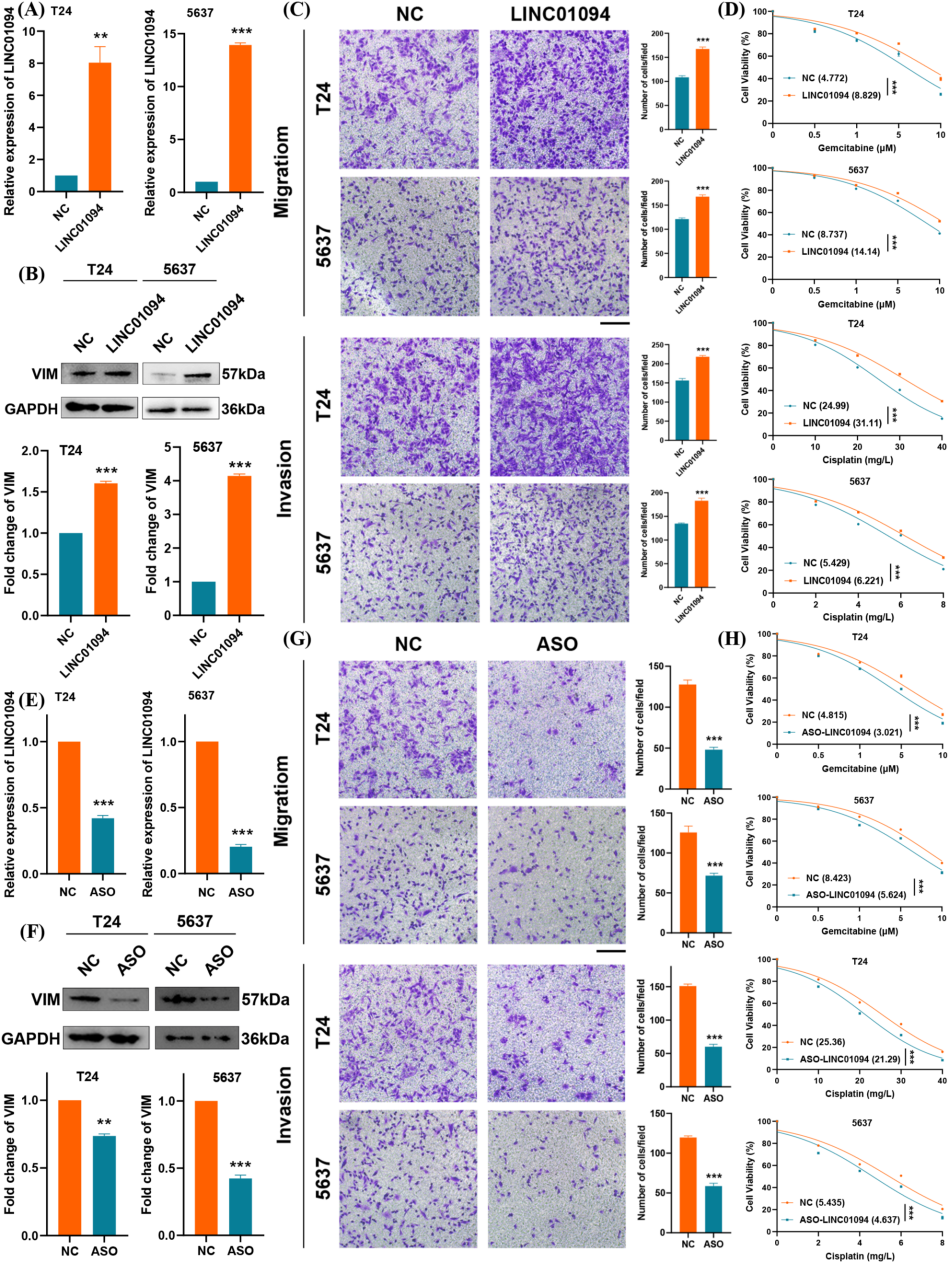
LINC01094 promotes BLCA metastasis and chemotherapy resistance. **(A)** qRT–PCR analysis of LINC01094 overexpression in BLCA cells. **(B)** Western blot showing elevated VIM protein upon LINC01094 overexpression. **(C)** Migration and invasion assays in BLCA cells overexpressing LINC01094. **(D)** CCK-8 assay evaluating gemcitabine and cisplatin sensitivity in cells overexpressing LINC01094. **(E)** qRT–PCR analysis of LINC01094 knockdown in BLCA cells. **(F)** Western blot showing reduced VIM protein under LINC01094 knockdown. **(G)** Migration and invasion assays in BLCA cells after LINC01094 knockdown. **(H)** CCK-8 assay evaluating drug sensitivity (gemcitabine/cisplatin) in cells with LINC01094 knockdown. **p< 0.01, ***p< 0.001. scale bar 100uM.

Guided by the above bioinformatic analyses, we explored LINC01094’s role in metastasis and chemotherapy resistance. Overexpression of LINC01094 in BLCA cell lines was confirmed by qRT-PCR (**Figure 9A**), and led to increased VIM protein expression (**Figure 9B**). Transwell migration and invasion assays demonstrated that LINC01094 significantly promoted the metastatic capacity of BLCA cells (**Figure 9C**). Furthermore, CCK-8 assays showed that LINC01094 overexpression enhanced cell viability under gemcitabine or cisplatin treatment (**Figure 9D**).

In contrast, stable knockdown of LINC01094 via ASOs effectively reduced its expression (**Figure 9E**) and decreased VIM protein levels (**Figure 9F**). As expected, silencing LINC01094 suppressed cell growth and migration in BLCA cells, as confirmed by the transwell assay (**Figure 9G**). Likewise, CCK-8 results indicated that LINC01094 knockdown significantly inhibited BLCA cell proliferation when exposed to gemcitabine or cisplatin (**Figure 9H**). Taken together, these data suggest that LINC01094 promotes metastasis and chemoresistance in BLCA, likely by stabilizing VIM. Targeting LINC01094 with ASOs may therefore represent a promising therapeutic strategy for BLCA.

## 4 Discussion

In this study, we systematically investigated the lactate-related gene signature in BLCA using consensus clustering analysis and constructed a lactate-based tSNE score with both prognostic and therapeutic predictive capabilities. Building on this scoring system, we identified LINC01094 as the gene most strongly correlated with the tSNE score. Correlation analyses further suggested that LINC01094 may be involved in immune- and inflammation-related signaling pathways, as well as tumor progression and metastasis pathways. We confirmed its expression in BLCA cell lines and tissues via qRT-PCR and FISH. Mechanistically, LINC01094 interacts with VIM, an essential molecule in the EMT process, thereby inhibiting ubiquitination and stabilizing VIM protein levels. Functionally, LINC01094 promoted both metastatic behavior and chemotherapy resistance in BLCA cells. We also established a lactate-based tSNE score that was strongly associated with overall survival and developed a nomogram validated by calibration curves. These findings may help personalize treatment options for BLCA.

Lactate was long regarded merely as a byproduct of aerobic glycolysis. However, accumulating evidence shows it can impact tumor responses to immune checkpoint therapy (21). Accordingly, a lactate-focused perspective on BLCA seems viable, especially given previous studies indicating that lactate-related gene signatures can effectively stratify colorectal and renal clear cell carcinomas (22, 23). Here, we undertook a comprehensive analysis of lactate-regulated pathways in BLCA, highlighting EMT as a key phenotype tied to metastasis and chemoresistance (24). Moreover, macrophage infiltration levels showed significant correlations not only with the tSNE score but also with LINC01094 expression. M2-like macrophages are known to suppress T-cell proliferation and differentiation while supporting tumor growth and metastasis (25–27). Our data indicates that LINC01094 may contribute to macrophage polarization in the BLCA TME, although this requires further experimental confirmation.

In recent years, the importance of lncRNAs in tumorigenesis and cancer progression has garnered increasing attention. LINC01094, also called CTEPHA1, was previously linked to chronic thromboembolic pulmonary hypertension (28) and has since been implicated in the malignant phenotypes of kidney cancer, glioma, and ovarian cancer (29–33). Yet, its role in BLCA remains largely unexplored. By examining public mass spectrometry datasets, we uncovered a molecular connection between LINC01094 and the EMT process, which we validated via *in vitro* experiments using an ASO approach. Notably, ASO-mediated knockdown of LINC01094 enhanced BLCA cell sensitivity to cisplatin and gemcitabine, paralleling earlier findings that other lncRNAs (e.g., FOXD2-AS1 and DLEU1) modulate chemoresistance via EMT-related mechanisms (34–37). Although our study primarily focused on *in vitro* evidence, future *in vivo* experiments, such as tumor xenograft models, are warranted to further evaluate the therapeutic efficacy of ASOs targeting LINC01094.

VIM is widely recognized for its roles in cell motility, mechanosensing, signal transduction, and inflammatory processes (38, 39), yet few studies have addressed how lncRNAs regulate VIM protein stability. One of our key findings is that LINC01094 elevates VIM protein levels, even though it also correlates with increased VIM mRNA. This suggests that LINC01094 influences both VIM transcription and post-translational stability. However, the precise molecular mechanisms—such as specific binding sites and whether additional cofactors are involved—remain to be elucidated. Further in-depth studies are thus needed to dissect how LINC01094 orchestrates VIM regulation in BLCA.

Nonetheless, the present study has several limitations that warrant careful consideration. Despite leveraging large-scale public datasets for our analyses, we acknowledge that these datasets may not fully capture the clinical and molecular heterogeneity of BLCA. Future multicenter prospective studies will be necessary to validate the generalizability of our findings (40).

Beyond LINC01094, other lncRNAs—including MALAT1, UCA1, and XIST, among others—have also been implicated in bladder cancer progression or metabolic reprogramming (41–43). Integrating multiple lncRNAs into a broader investigational framework could yield a more comprehensive view of how non-coding RNAs co-regulate lactate metabolism, immune interactions, and chemoresistance in BLCA.

While our *in vitro* approach clarified several key mechanistic aspects, *in vivo* models—such as orthotopic implants or patient-derived xenografts—could better recapitulate the complex tumor microenvironment and more accurately reflect clinical conditions (44).

We have not yet performed direct co-culture or single-cell assays to observe how lactate metabolism affects specific immune cell populations (e.g., Tregs, M2 macrophages) within the tumor microenvironment. Such functional assays are crucial for delineating the direct interplay between lactate-driven pathways and immune cell phenotypes (9).

Lastly, our current work did not measure lactate levels or flux directly. Incorporating stable-isotope tracing and quantitative assays for lactate production and consumption will be an important next step to validate how metabolic alterations influence tumor progression and immune responses in BLCA (45–47).

## 5 Conclusions

In conclusion, our research offers new insights into lactate-related gene signatures in BLCA and their impact on the tumor microenvironment and patient prognosis. We developed a lactate-based tSNE score capable of identifying patients at higher risk and predicting responses to immunotherapy and chemotherapy. Furthermore, our work demonstrates that LINC01094 promotes metastasis and chemoresistance in BLCA cells by stabilizing VIM protein, indicative of an EMT-related pathway. Targeting LINC01094 with antisense oligonucleotides (ASOs) could represent a promising therapeutic strategy, meriting additional exploration *in vivo*. Ultimately, our findings could lead to the identification of novel biomarkers and treatment targets, thereby advancing precision medicine in BLCA.

## Data Availability

The data analyzed in this study were obtained from the publicly accessible TCGA-BLCA project in the NCI Genomic Data Commons (https://portal.gdc.cancer.gov/projects/TCGA-BLCA. No new RNA-seq data were generated in this study.
