# Supplementary figures and images for: A lactate-related tSNE signature defines prognostic subtypes of bladder cancer and reveals LINC01094-mediated VIM stabilization in metastasis and drug resistance
Source: Front Immunol. 2025 May 14;16:1593523. doi: 10.3389/fimmu.2025.1593523 (PMC12116251; doi:10.3389/fimmu.2025.1593523)

# NMF rank survey

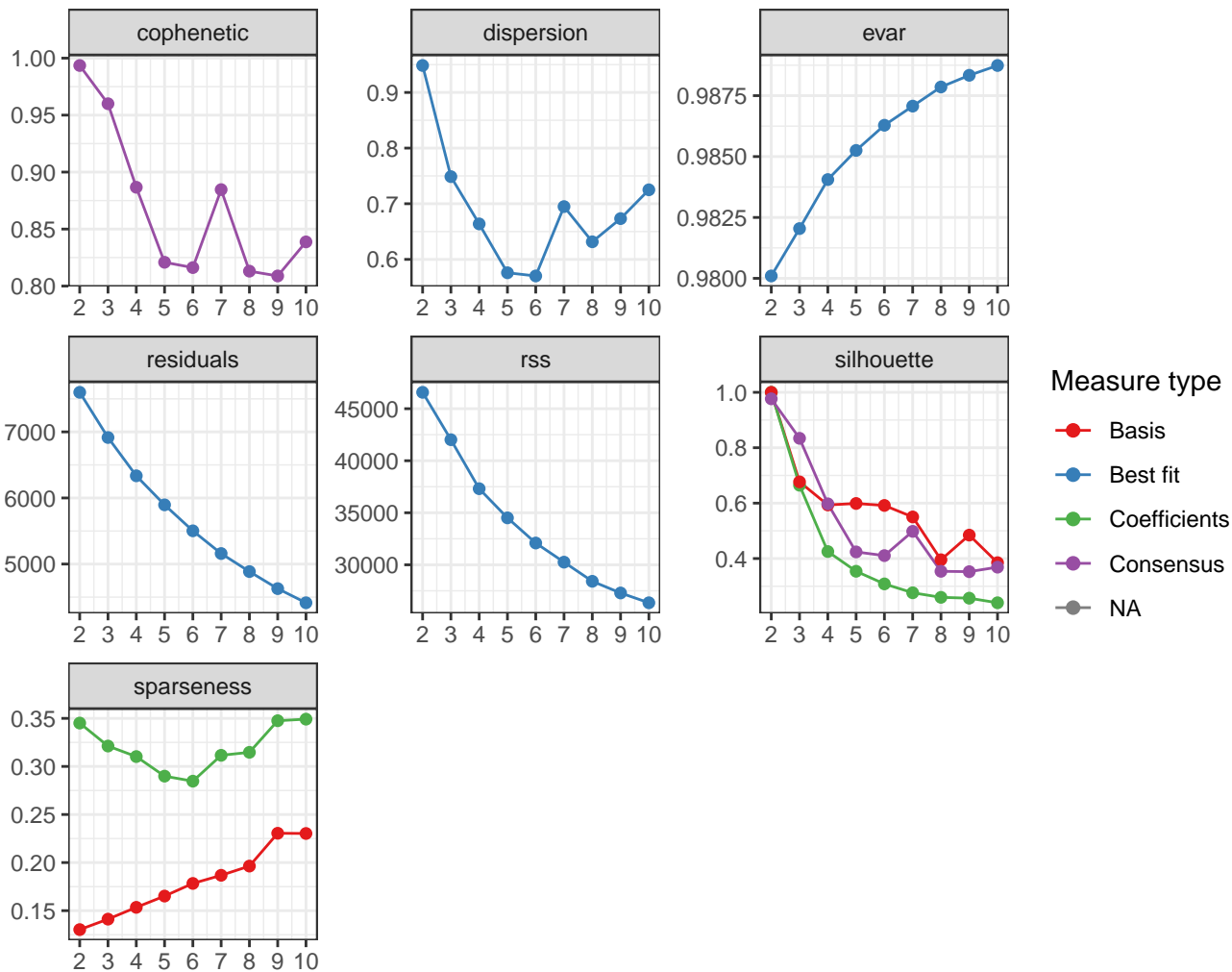

Factorization rank

Supplement: Supplementary file 1 [file Image1.pdf]

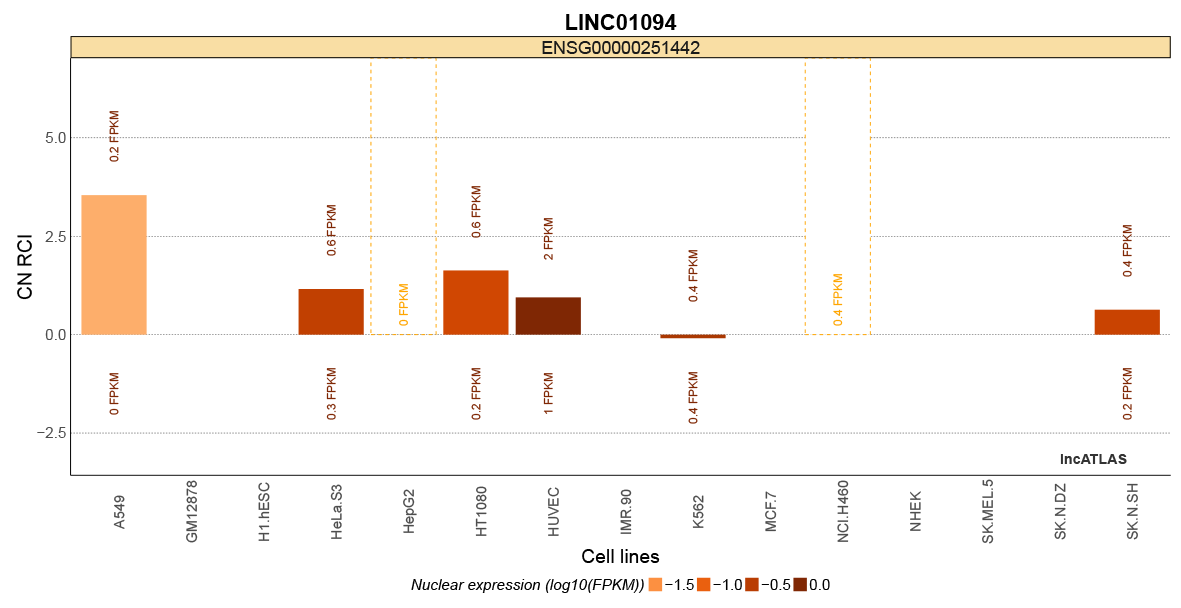

Supplement: Supplementary file 2 [file Image2.tif]
